# Supplementary material for: Pushing of Magnetic Microdroplet Using Electromagnetic Actuation System
Source: Nanomaterials (Basel). 2020 Feb 20;10(2):371. doi: 10.3390/nano10020371 (PMC7075344; doi:10.3390/nano10020371)
Supplement: Supplementary file 1 [file nanomaterials-10-00371-s001.pdf]

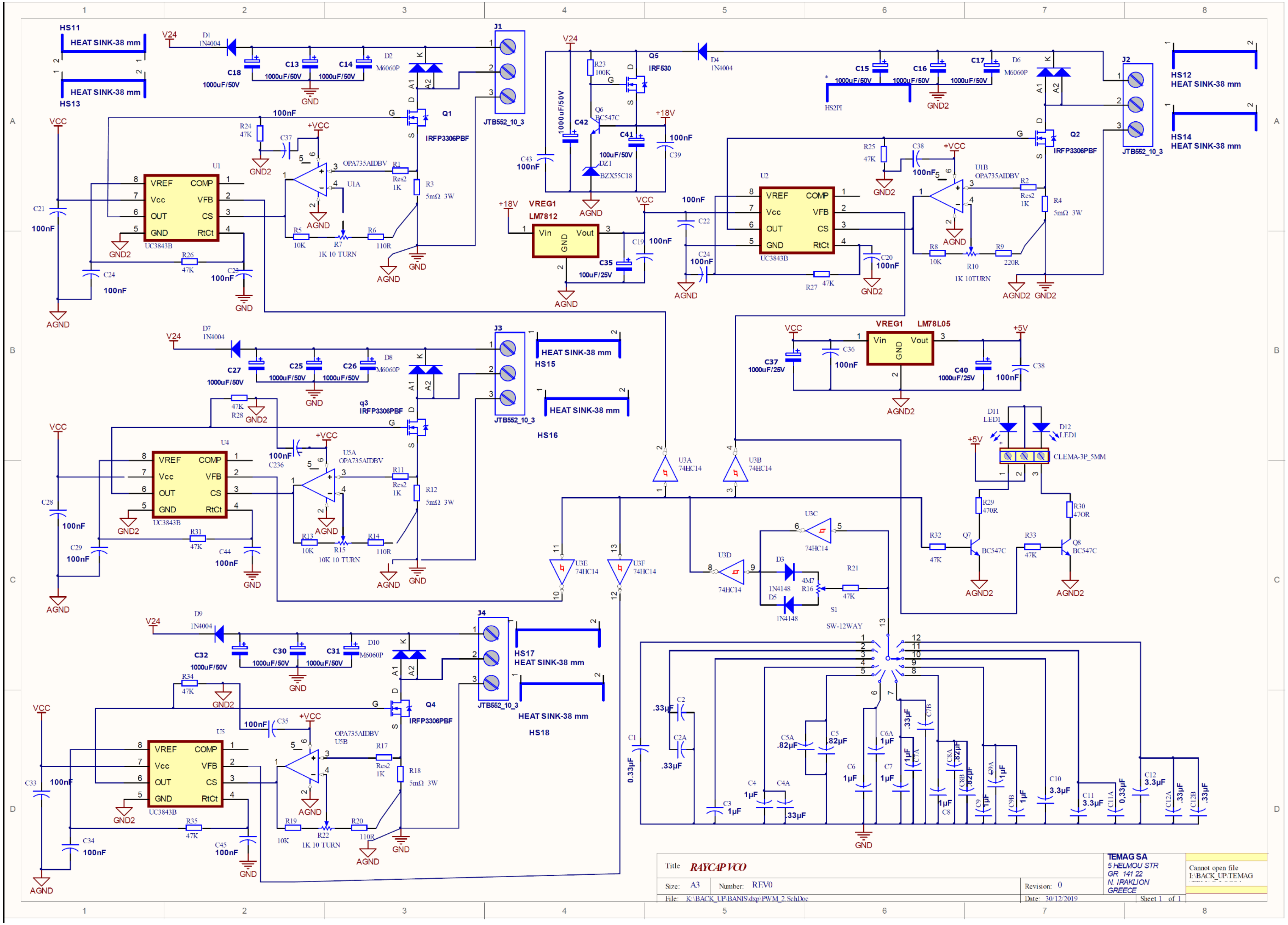

HS11
HEAT SINK-38 mm
HS13
V24
D1
1N4004
C18
1000uF/50V
C13
C14
D2
M6060P
J1
JTB552_10_3
GND
Q1
IRFP3306PBF
100nF
VCC
+VCC
R24
47K
C37
GND2
U1
OPA735AIDBV
U1A
R1
Res2
1K
R3
5mΩ 3W
VREF
COMP
Vcc
VFB
OUT
CS
GND
RtCt
UC3843B
C21
R26
47K
C23
C24
R5
10K
R7
1K 10 TURN
R6
110R
AGND
V24
R23
100K
Q5
IRF530
Q6
BC547C
C42
C41
100uF/50V
DZ1
BZX55C18
+18V
C39
C43
1000uF/50V
D4
1N4004
VREG1
LM7812
Vin
Vout
C19
C35
100uF/25V
C22
C15
C16
C17
D6
M6060P
HS2PI
GND2
J2
JTB552_10_3
HS12
HEAT SINK-38 mm
HS14
Q2
IRFP3306PBF
R25
47K
C38
U1B
OPA735AIDBV
R2
Res2
1K
R4
5mΩ 3W
U2
UC3843B
R8
10K
R10
1K 10TURN
R9
220R
C20
R27
47K
AGND2
D7
1N4004
J3
HS15
HS16
C27
C25
C26
D8
M6060P
q3
IRFP3306PBF
R28
47K
U4
U5A
OPA735AIDBV
C236
R11
Res2
1K
R12
5mΩ 3W
C28
C29
R31
47K
C44
R13
10K
R15
10K 10 TURN
R14
110R
U3A
74HC14
U3B
74HC14
VREG1
LM78L05
+5V
C37
1000uF/25V
C36
C40
1000uF/25V
D11
LED1
D12
LED1
CLEMA-3P_5MM
R29
470R
R30
470R
U3C
74HC14
U3D
U3E
U3F
D3
D5
1N4148
R16
4M7
R21
47K
S1
SW-12WAY
R32
47K
Q7
BC547C
R33
47K
Q8
BC547C
D9
1N4004
J4
HS17
HEAT SINK-38 mm
HS18
C32
C30
C31
D10
M6060P
Q4
IRFP3306PBF
R34
47K
U5
U5B
OPA735AIDBV
R17
Res2
1K
R18
5mΩ 3W
C33
C34
R35
47K
C45
R19
10K
R22
1K 10 TURN
R20
110R
C1
0.33µF
C2
.33µF
C2A
C3
1µF
C4
C4A
C5
.82µF
C5A
C6
C6A
C7
C7A
C7B
C8
C8A
C8B
C9
C9A
C9B
C10
3.3µF
C11
C11A
C12
C12A
C12B
TEMAG SA
5 HELMOU STR
GR 141 22
N. IRAKLION
GREECE
Title RAYCAP VCO
Size: A3
Number: REV0
Revision: 0
File: K:\BACK_UP\BANIS\dxp\PWM_2.SchDoc
Date: 30/12/2019
Sheet 1 of 1
Cannot open file
I:\BACK_UP\TEMAG
